# Influence of Key Processes on the Aroma Formation of Cicada Black Tea

**DOI:** 10.3390/foods15020401

**Published:** 2026-01-22

**Authors:** Xueke Li, Qiaoyi Zhou, Chengying Ma, Hongling Xia, Dongxia Liang, Aiqing Miao, Fanrong Cao, Caijin Ling

**Affiliations:** 1 Guangdong Provincial Key Laboratory of Tea Plant Resources Innovation and Utilization, Tea Research Institute, Guangdong Academy of Agricultural Sciences, Guangzhou 510640, China; li0309@stu.scau.edu.cn (X.L.); zhouqyi@foxmail.com (Q.Z.); machengying@tea.gdaas.cn (C.M.); xiahongling@gdaas.cn (H.X.); liangdx3407@163.com (D.L.); aiqing-miao@163.com (A.M.); 2 Engineering Technology Research Center of Guangdong Southern Characteristic Tea, College of Horticulture, South China Agricultural University, Guangzhou 510640, China

**Keywords:** *Empoasca onukii* Matsuda, black tea, GC-MS, volatile compounds

## Abstract

Cicada black tea is made from *Empoasca onukii*-infested tea leaves. The biochemical differences between Cicada black tea and regular black tea, and the dynamic changes in volatile components during processing, remain unclear. This study focused on the withering and fermentation stages, which most significantly affect the quality of black tea, to systematically investigate cicada black tea aroma formation. Ninety volatile compounds were identified, among which 12 key volatiles significantly contributed to the aroma characteristics. During withering, floral and fruity aromas were enhanced. Fermentation primarily intensified honey-like and sweet notes, which were mainly regulated by carotenoid-derived volatiles with low odor thresholds, such as β-Damascenone. Withering to a moisture content of 55% (17 h) and fermenting for 5 h comprised the optimal processing parameters to achieve the best aroma attributes. These insights into the mechanism of Cicada black tea aroma formation will lead to improved processing techniques to enhance its unique flavor.

## 1. Introduction

Tea (*Camellia sinensis* (L.) *O. Kuntze*) is one of the most economically important crops globally, with a long history of cultivation and consumption, as well as centuries of development. Among them, black tea is the most widely consumed worldwide, accounting for approximately 75% of global tea consumption [1]. It derives its distinctive sweet, floral, and honey-like aroma profile from intricate biochemical transformations during processing, particularly withering and fermentation [2,3]. Among the various types of black tea, Cicada black tea (a distinctive black tea produced from tea leaves infested by the tea green leafhopper, *Empoasca onukii* Matsuda) stands out for its unique flavor profile, characterized by a rich, sweet, and honey-like aroma, which is attributed to the infestation of tea leaves by *E. onukii* Matsuda. This infestation induces the production of specific secondary metabolites in the tea plant, imparting the leaves with a distinctive aroma and taste [4].

Black tea processing hinges critically on withering and fermentation for aroma and taste development: withering initiates leaf dehydration, triggering breakdown of proteins, carbohydrates, and lipids to generate volatile compound (VC) precursors [5,6]. Fermentation then oxidatively transforms these precursors into key aroma-active compounds (e.g., alcohols, aldehydes, and ketones) [7,8], with dynamic VC changes during these stages being essential for characteristic black tea flavor formation [9,10].

Notably, the processing techniques associated with *E. onukii* infestation have been classically applied in oolong tea production, such as Taiwan’s “Oriental Beauty Oolong Tea,” renowned for its honey-like sweetness and complex floral notes [11]. The core aroma compounds in such teas include terpenoids like 2,6-dimethyl-3,7-octadiene-2,6-diol [12,13]. However, research on applying this biotic stress-induced process to black tea production (i.e., Cicada black tea) is still in its infancy.

Recent studies on black tea processing primarily focused on conventional black tea, with limited attention given to the unique processing dynamics of Cicada black tea. Although research has explored the effects of withering and fermentation on the aroma characteristics of conventional black tea [8,14], the synergistic effects of these processes on *E. onukii*-infested tea leaves remain understudied. This knowledge gap hinders the optimization of Cicada black tea processing and limits its quality improvement and economic potential.

Therefore, the present study aims to address this gap by systematically investigating the dynamic changes in VCs during the withering and fermentation processes of Cicada black tea. Specifically, the objectives of this study include (1) analyzing the dynamic changes in the content of VCs during withering and fermentation; (2) identifying key aroma-active compounds and their precursors in Cicada black tea; and (3) determining the impact of different processing stages on the formation of characteristic flavors. By comprehensively revealing the mechanisms underlying aroma formation in Cicada black tea, this study provides a critical foundation to optimize processing parameters and enhance the quality of this unique tea variety. This study not only deepens our understanding of the biochemical processes behind the unique flavor of Cicada black tea but also offers practical guidance to improve its processing techniques. By identifying key aroma compounds and their formation pathways, this research lays the groundwork for future innovations in tea processing, ultimately contributing to the enhanced economic value and market competitiveness of Cicada black tea.

## 2. Materials and Methods

### 2.1. Experimental Materials

The tea plants of the Jinxuan cultivar were cultivated in the field at the Tea Research Institute of the Guangdong Academy of Agricultural Sciences (Yingde, China). On 5 June 2023, approximately 75 kg of injured tender shoots from plants infested by *E. onukii* and 75 kg of healthy tender shoots from uninfested plants (each shoot consisting of one bud and two leaves) were collected. The fresh leaves infested by *E. onukii* exhibited a light green to yellowish color, shortened internodes, and red spots on the stems and leaves, while the uninfested fresh leaves were green without red spots. The fresh leaves infested by *E. onukii* (CY) and the uninfested leaves (CK) were microwave-fixed in a 700 W microwave oven for 2 min, manually flipped, microwaved for an additional 2 min to ensure uniform enzyme inactivation, and then stored at −80 °C as reference samples.

### 2.2. Black Tea Production

The collected leaves were transferred to the production workshop and evenly spread on open withering troughs. The leaves were divided into three groups: those withered to 65% moisture content for 10 h (W65); those withered to 60% moisture content for 14 h (W60); and those withered to 55% moisture content for 17 h (W55). During the withering process, the ambient temperature was maintained at 25–27 °C, with a relative humidity of 65–70%. Each replicate sample consisted of 25 kg of leaves, and each withering group (W) had three replicate samples. The withered leaves from these three treatments were then transferred to a fermentation room, where they were fermented at a constant temperature of 25 °C for 3, 5, or 7 h, respectively. Each fermentation group (F) consisted of three replicate samples, with each replicate containing 5 kg of leaves. The uninfested tea leaves were withered to 65% moisture content and then fermented for 5 h (CK-WF). Fresh, withered, and fermented samples were microwave-fixed at 700 W for 2 min, turned over, microwave-fixed again at 700 W for 2 min, dehydrated using a hot air dryer at 60 °C for 4 h to constant weight, and stored at 80 °C for further analysis.

### 2.3. Volatile Compound (VC) Extraction and Identification

#### 2.3.1. Aroma Extraction Method

All tea samples were ground into powder at room temperature (25 °C). Precisely 1.000 ± 0.005 g of each sample was weighed into a 20 mL headspace vial, followed by the addition of 2 μL of internal standard (ethyl decanoate in n-hexane solution, concentration: 100 μg/mL) prior to analysis. For extraction, the headspace vial was first placed in a heating agitator and equilibrated at 60 °C for 5 min. Subsequently, extraction was performed using an SPME Arrow fiber (Agilent, Santa Clara, CA, USA) at 60 °C for 40 min. After completion, the sample was analyzed using chromatography–mass spectrometry (GC-MS).

#### 2.3.2. GC-MS Conditions

An HP-5MS elastic quartz capillary column (30 m × 0.25 mm, internal diameter 0.25 µm; Agilent Technologies; Santa Clara, CA, USA) was used. The temperature program was set according to the method described by [15]. After injection, the sample was desorbed at 230 °C for 5 min. The MS ionization mode was electron impact (EI), the ion source temperature was 230 °C, and the electron energy was 70 eV. The scanning range was 35–450 u. High-purity helium (He, purity > 99.999%) was used as the carrier gas at a flow rate of 1 mL/min in splitless mode. The electron multiplier voltage was set to 1800 V, and the total ion current intensity was 100 mA.

#### 2.3.3. Qualitative and Quantitative Analysis of Volatile Compounds

Qualitative analysis of the mass spectra was performed using the NIST standard mass spectral library in the Xcalibur workstation (Thermo Scientific, Waltham, MA, USA) and relevant literature. For the quantitative analysis, 2 µL of a 100 µg/mL ethyl decanoate in n-hexane solution was added to the tea sample as an internal standard. The relative content of VCs was calculated based on the internal standard. Each tea sample was analyzed in triplicate.

### 2.4. Statistical and Multivariate Analysis

The average values and standard errors of all parameters were calculated from three technical replicates. Partial Least Squares Discriminant Analysis (PLS-DA) and Orthogonal PLS-DA (OPLS-DA) were performed using SIMCA-14.2 software (Umetrics, Umea, Sweden). Multiple comparisons were conducted using one-way analysis of variance (ANOVA) with SPSS statistical software version 20.0 (IBM Corp., Armonk, NY, USA). Bar charts were generated using Origin 2022 software (OriginLab Corporation, Northampton, MA, USA), and Venn diagrams were created using the website https://bioinformatics.psb.ugent.be/webtools/Venn/ (accessed on 18 January 2025).

### 2.5. Sensory Evaluation of Tea Aroma

Referring to the Chinese Academy of Agricultural Sciences by following the Methodology of Sensory Evaluation of Tea (GB/T 23776-2018) [16], four tea evaluation experts were invited to evaluate the tea aroma quantitatively. The specific steps were as follows: 3.0 g of tea samples were added to 150 mL of pure boiling water. After brewing for 5 min, the tea and water were separated immediately. The experts evaluated and scored the infused tea aroma at the cup bottom. The aroma of tea samples was scored across five dimensions: “floral aroma, sweet aroma, green grass aroma, honey aroma, and fruity aroma”. During the evaluation process, panelists rated the samples on a scale of 0 to 5 points.

### 2.6. Odor Activity Value (ROAV) Analysis

OAV analysis is commonly used to evaluate the contribution of aroma compounds. Aroma compounds with an ROAV > 1 are generally considered to contribute significantly to the overall aroma characteristics. The calculation formula for the ROAV was as follows:

ROAV = Ci/OTi.(1)

where OTi represents the threshold value of the compound in water and Ci is the relative concentration of the volatile components.

## 3. Results and Discussion

### 3.1. Comparative Analysis of Volatile Compounds in Fresh Leaves of Cicada Black Tea and Regular Black Tea

Using headspace solid-phase microextraction coupled with gas chromatography–mass spectrometry (HS-SPME-GC-MS), this study systematically analyzed the differences in VCs between fresh leaves of Cicada black tea (infested by the tea green leafhopper *E. onukii* Matsuda) and regular black tea, with a focus on the impact of *E. onukii* infestation on VC profiles.

Based on OPLS-DA, fresh leaf samples of the two tea types showed significant separation in volatile composition (model parameters: R^2^ = 0.999, Q^2^ = 0.999), indicating that *E. onukii* infestation significantly altered the aroma metabolic profiles of fresh leaves (Figure S1). As shown in Figure S2, all 200 permutated samples were distributed below the original model (validation parameters: R^2^ = 0.265, Q^2^ = −0.762), confirming the robustness of the model. Fresh leaf samples of Cicada black tea clustered together, distinctly separated from those of regular black tea, demonstrating unique aroma characteristics between the two varieties at the fresh leaf stage.

**Figure 1:**
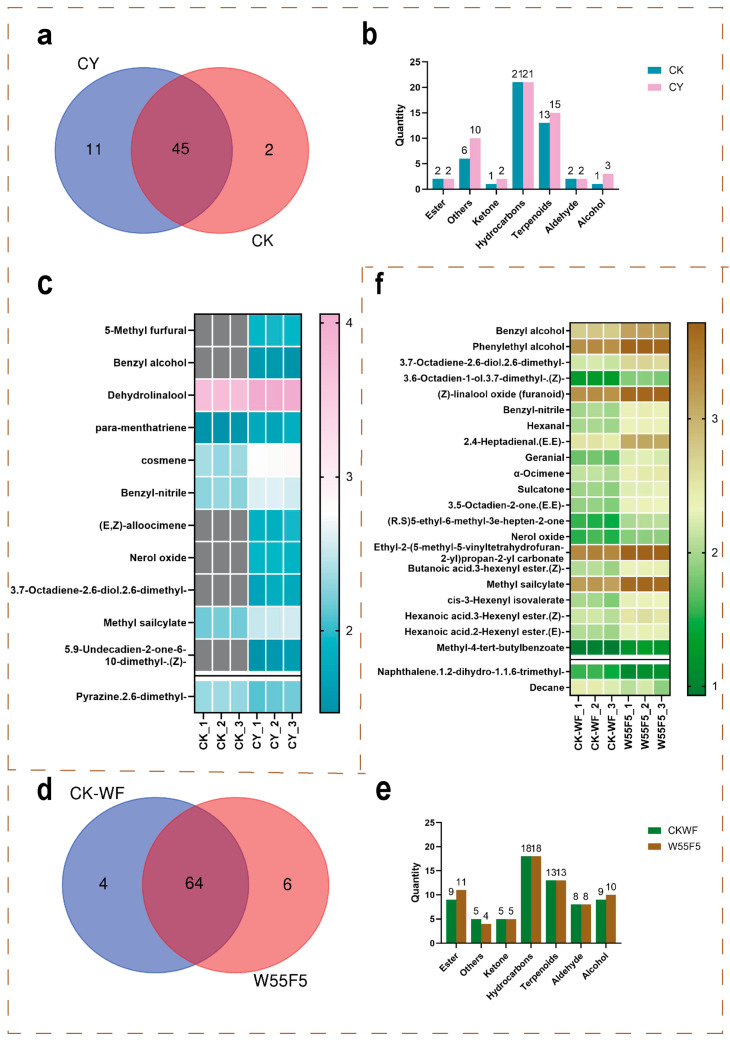
Volatile compound analysis of Cicada black tea and conventional black tea in Fresh Leaves and Finished Tea Products. (**a**,**d**) Venn diagrams showing differentially abundant compounds between Cicada black tea and conventional black tea in fresh leaves and finished tea products. (**b**,**e**) Analysis of the quantitative differences in seven categories of differentially abundant compounds between Cicada black tea and conventional black tea in fresh leaves and finished tea products. (**c**,**f**) Heatmaps of differentially abundant volatile compounds with fold change (FC) > 2 or <0.5 between Cicada black tea and conventional black tea in fresh leaves and finished tea products. (W followed by numbers indicates withering degree, i.e., leaf moisture percentage; F followed by numbers denotes withering duration in hours). CY, fresh leaves infested by *E. onukii*; CK, uninfested leaves.

A total of 57 VCs were identified in the fresh leaves of both tea types. Among them, 11 unique compounds were detected in the fresh leaves of Cicada black tea, including benzyl alcohol, 2,6-dimethyl-3,7-octadiene-2,6-diol, and 2,6-dimethyl-2,4,6-octatriene (Figure 1a). Figure 1c presents 12 differentially abundant compounds screened through volcano plot analysis, of which 11 were upregulated in the fresh leaves of Cicada black tea compared with those in regular black tea, while 1 was downregulated. The concentrations of terpenoids (e.g., linalool, geraniol) and other compounds (e.g., nerol oxide) were significantly upregulated (fold change (FC) > 2, *p* < 0.1). Their sensory characteristics (e.g., floral, fruity, and honey-like aromas) correlated highly with the aroma profile of the finished Cicada black tea. In contrast, the content of 2,6-dimethylpyrazine, which exhibits a roasted aroma, was significantly lower in the fresh leaves of Cicada black tea than in regular black tea (FC < 0.5, *p* < 0.1). Overall, the fresh leaves of Cicada black tea showed an upregulation of floral, fruity, and sweet aroma compounds and a downregulation of roasted aroma compounds, resulting in a fresher and more vibrant aroma profile.

The VCs in the samples were classified into seven categories: esters, ketones, hydrocarbons, terpenoids, aldehydes, alcohols, and others. A total of 55 VCs were detected in the fresh leaves of Cicada black tea, while 46 were detected in regular black tea. After *E. onukii* infestation, both the number and content of VCs in the fresh tea leaves increased. In the fresh leaves of Cicada black tea, the categories of “others,” ketones, terpenoids, and alcohols showed significant increases (Figure 1b).

Studies have shown that *E. onukii* infestation can activate secondary metabolic pathways in tea plants, promoting the expression of terpene synthase genes (e.g., *CsTPS*), thereby increasing the accumulation of monoterpenes and sesquiterpenes [4]. Additionally, terpenoids (e.g., linalool) are the primary contributors to the characteristic honey-like aroma of Oriental Beauty oolong tea [17]. The content of benzyl alcohol in the fresh leaves of Cicada black tea was significantly higher than that in the control group (Figure 1d), and its sweet and honey-like aroma characteristics are important contributors to the aroma of Cicada black tea [12]. It was found that benzyl alcohol is a key metabolite in the defense response of tea plants after *E. onukii* infestation. The content of benzyl alcohol correlated positively with the degree of *E. onukii* infestation, and its synthesis is directly related to the enhanced activity of phenylalanine decarboxylase. Furthermore, 2,6-dimethyl-3,7-octadiene2,6-diol has been confirmed as a biomarker of pest stress [4], with an FC value of 5.73. This compound is produced through the enzymatic oxidation of linalool, and its presence further supports the uniqueness of the aroma metabolism in the fresh leaves of Cicada black tea. Notably, although indole in the fresh leaves of Cicada black tea did not reach the FC threshold, its relative content was still 1.2 times higher than that in the control group [18], demonstrating that *E. onukii* infestation can induce the overexpression of tryptophan synthase genes (*CsTSA/CsTSB2*) in tea plants, thereby promoting the synthesis of indole. This mechanism might explain the observed increase in indole content in the fresh leaves of Cicada black tea in this study. (E,Z)-alloocimene was found to attract stink bugs in a study on their antennal response. We speculate that the synthesis of (E,Z)-alloocimene in tea plants serves as an indirect defense strategy aimed at attracting natural enemies of the tea green leafhopper [19]. Research by [20] demonstrated that the expression of the terpene synthase CsTPS08 is significantly induced in response to herbivory, such as feeding by Spodoptera litura. This enzyme catalyzes the production of a mixture dominated by linalool, along with other terpenes—including β-myrcene, α-terpinolene, and (E,Z)-alloocimene—which play dual roles in defending against pests and diseases and in shaping the aroma quality of tea.

In summary, *E. onukii* infestation significantly altered the VC metabolic profile of fresh tea leaves, with terpenoids, alcohols, and other compounds being the key differential components. The unique terpenoids (e.g., linalool, geraniol) and other compounds (e.g., nerol oxide) in the fresh leaves of Cicada black tea are the primary sources of its floral, fruity, and honey-like aroma characteristics. Meanwhile, benzyl alcohol and 2,6-dimethyl-3,7-octadiene-2,6-diol, as biomarkers of pest stress, further validate the uniqueness of its aroma metabolism.

### 3.2. Comparative Analysis of Volatile Compounds in Cicada Black Tea and Conventional Black Tea

To further investigate the impact of *E. onukii* infestation on the VCs of black tea, OPLS-DA was applied to statistically analyze the VCs identified in two types of finished black tea samples differentiated by the presence or absence of *E. onukii* infestation.

Based on the OPLS-DA results (Figure S3), the replicate groups of the two tea samples clustered together, and a clear separation was achieved between the two groups (model parameters: R^2^ = 0.998, Q^2^ = 0.991). As shown in Figure S4, all 200 permutated samples were distributed below the original model (validation parameters: R^2^ = 0.595, Q^2^ = −0.496), confirming the robustness of the model. Samples of Cicada black tea clustered together, being separated distinctly from those of regular black tea, thus indicating that the two types of black tea exhibit different aroma characteristics.

Among the 73 VCs detected, six unique compounds were identified in Cicada black tea, including pentadecane, 1-octanol, and cis-3-hexenyl-α-methylbutyrate. In contrast, four unique compounds, such as undecane, were detected in regular black tea (Figure 1d). Additionally, the distribution of the seven major categories of VCs in the two finished black teas showed significant differences. Regular black tea contained a higher number of compounds in the “others” category, while Cicada black tea had a higher proportion of esters and alcohols (Figure 1e). The differentially abundant compounds between the two black teas were primarily esters and hydrocarbons. Esters are important contributors to the aroma profile of black tea [21], and the distinct ester compositions in Cicada black tea and regular black tea result in their unique aroma characteristics.

Using volcano plot analysis with the criteria of FC > 2 or <0.5 and *p* < 0.1, a total of 23 differentially abundant VCs were identified (Figure 1f). Among them, 21 compounds were significantly upregulated in Cicada black tea (FC > 2), while two compounds (1,2-dihydro-1,1,6-trimethylnaphthalene, FC = 0.28; decane, FC = 0.48) were downregulated. Notably, 20 of these compounds have been reported to contribute significantly to aroma. The ROAV is a measure used to assess the contribution of volatile compounds to food flavor [22]. Among the 14 VCs discussed below, significant contributions or modifying effects on the tea aroma profile during the withering process were observed (Table S2). Compounds with an ROAV > 1 are generally considered to make a significant contribution to aroma. For example, (E,E)-2,4-heptadienal, which exhibits nutty, violet, watermelon, and grassy aromas, was present at significantly higher concentrations in Cicada black tea than in regular black tea (Table 1), making it an important contributor to the dominant aroma characteristics.

Additionally, key VCs can be classified into honey-like and sweet aroma types. Honey-like aroma compounds include benzyl alcohol and phenylethyl alcohol, with the total ROAV of honey-like compounds in Cicada black tea being 2.36 times higher than that in regular black tea. Sweet aroma compounds include (Z)-linalool oxide (furanoid), geranial, and (Z)-3,7-dimethyl-3,6-octadien-1-ol, with the total ROAV of sweet aroma compounds in Cicada black tea being 2.35 times higher than that in regular black tea. Furthermore, Ref. [13] was observed that *E. onukii* infestation significantly increased the content of Ethyl-2-(5-methyl-5-vinyltetrahydrofuran-2-yl)propan-2-yl carbonate in oolong tea. In this study, the abundance of this ester compound was also significantly upregulated in Cicada black tea (FC = 2.05, *p* < 0.05), with a concentration of 5105.02 ± 137.54 μg/kg (W55F5). This compound exhibits a fruity aroma, and although its odor threshold has not been reported, its high relative content suggests that it might contribute to the unique aroma characteristics of Cicada black tea, thereby enhancing its fruity complexity.

The honey-like and sweet aroma characteristics further highlight the enhancing effect of pest stress on aroma quality. Notably, the contents of geraniol, linalool, and linalool oxides in cicada black tea were higher than those in regular black tea. This might be attributed to the significant increase in β-glucosidase activity during the late withering stage, which promotes the conversion of glycosides into terpenoid VCs, thereby increasing the levels of geraniol, linalool, and linalool oxides [23,24]. However, since β-glucosidase activity was not directly measured in this study, this relationship remains a correlation rather than a confirmed mechanism.

In summary, Cicada black tea, produced from *E. onukii*-infested fresh leaves, exhibits significant differences in aroma compared with regular black tea. Its unique aroma profile is primarily composed of (E,E)-2,4-heptadienal, phenylethyl alcohol, and linalool oxides, complemented by the synergistic effects of alcohols and esters. These findings not only reveal the long-term impact of pest stress on the aroma components of black tea but also provide key metabolic markers to optimize the processing of Cicada black tea.

### 3.3. Comparative Analysis of Volatile Compounds in Cicada Black Tea Under Different Withering Conditions

To investigate the impact of withering conditions on the aroma formation of Cicada black tea, this study analyzed the differences in VCs among tea samples withered to three different moisture levels (55%, 60%, and 65%) using a PLS-DA model.

The PLS-DA results revealed significant separation in the metabolic profiles of the different withering treatment groups (R^2^Y = 0.987, Q^2^ = 0.943), indicating that the degree of withering significantly regulates VCs. The scores exhibited distinct spatial separation during the withering process. Specifically, fresh leaves were located in the fourth quadrant, while samples with 65% moisture content and 60% moisture content were positioned in the second quadrant, and samples with 55% moisture content were placed in the third quadrant. The closer the spatial distance, the smaller the changes in volatile metabolites. Therefore, significant changes in VCs occurred after the onset of fermentation, and the differences in VCs between fresh leaves and withered leaves became more pronounced as withering progressed (Figure S5). As shown in Figure S6, all 200 permutated samples were distributed below the original model, with parameters (R^2^ = 0.18, Q^2^ = −0.422) indicating strong robustness of the model.

**Figure 2:**
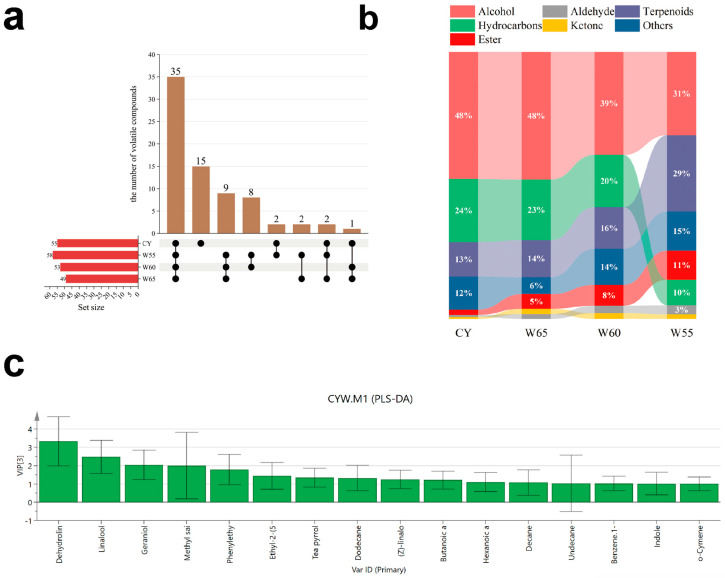
Screening and analysis of representative volatile components during the withering process of Cicada black tea. (**a**) Upset plot of CY, W65, W60, and W55 samples. The black dots in the cross-matrix below represent volatile compounds shared among different sample combinations. (**b**) Dynamic changes in seven categories of volatile compounds analyzed by gas chromatography–mass spectrometry during processing from CY to W55 (CY: fresh leaves; W followed by numbers indicates the withering degree, i.e., leaf moisture percentage). (**c**) Sixteen major volatile compounds with variable importance in projection (VIP) > 1. PLS-DA, Partial Least Squares Discriminant Analysis.

Among the 75 identified VCs, 55 were detected in fresh leaves, including 15 unique compounds such as β-myrcene (Figure 2a). Most of the compounds unique to fresh leaves exhibited roasted or baked aromas, and their reduction allows the floral and fruity aromas of the tea to become more prominent (Table S1). As the withering time increased (the moisture content decreased from 65% to 55%), the number of VCs first decreased and then increased, indicating that some VCs underwent transformation. During the mid-to-late stages of withering (W60, W55), eight unique compounds emerged, including 1-hexanol and (E)-3-hexen-1-ol, with three alcohols and three esters among them (Table S2). Most of the compounds that appeared during the mid-to-late withering stages exhibited floral, fruity, and fresh green aromas. Withering increased the content of floral and fruity compounds; however, the content of grassy and green aromas also increased with the degree of withering [25]. As withering progressed, the relative contents of terpenoids and other compounds continued to increase, while the relative contents of alcohols and hydrocarbons significantly decreased (Figure 2b). This trend was closely related to enzymatic reactions (e.g., glycoside hydrolysis, lipid oxidation) triggered by dehydration during withering [14].

In summary, the content of VCs in the leaves continuously increased as withering progressed. These results align with previous studies, demonstrating that the total VC content increases with prolonged withering time [14,26].

Sixteen compounds had variable importance in projection (VIP) values greater than 1 (Figure 2c), with dehydrolinalool, geraniol, and linalool being the most significant contributors. Dehydrolinalool was most abundant in fresh leaves, reaching 11,084.25 ± 391.86 μg/kg, and its content gradually decreased as withering progressed. Geraniol reached a content of 932.9 ± 69.31 μg/kg (ROAV = 141.35) in tea samples withered to 55% moisture content, representing a 6.1-fold increase compared with samples with 65% moisture content (152.89 ± 8.52 μg/kg). Its rose-like aroma became more pronounced with increasing withering degree (Table S2). Linalool reached a content of 1952.73 ± 195.81 μg/kg (ROAV = 325.46) in tea samples with 55% moisture content, representing a 2.78-fold increase compared with fresh leaves, indicating that the withering process effectively promoted the release and transformation of terpenoid precursors [27]. Additionally, (Z)-3-hexenyl butanoate was detected after the onset of withering, and its fresh, sweet, and fruity aroma characteristics added a unique complexity to Cicada black tea.

Notably, indole, phenylethyl alcohol, and (Z)-linalool oxide (furanoid) exhibited dynamic changes during the withering process. Indole was present at high levels in fresh leaves, sharply decreased during the early withering stage (65% moisture content), but rebounded at 55% moisture content. This phenomenon might be related to enhanced tryptophan metabolism resulting from accelerated protein degradation during the late withering stage [18]. The relative content of phenylethyl alcohol was below the detection limit in fresh leaves and during the early withering stage (65% moisture content); however, it increased to 595.48 ± 58.28 μg/kg at 55% moisture content. Its rose-like and honey-like aroma characteristics contributed to the dominant honey and floral notes of Cicada black tea. The relative content of (Z)-linalool oxide (furanoid) increased by 3.26-fold as withering progressed, adding sweetness to Cicada black tea.

Glycoside-derived volatiles (GDVs), which contribute to sweet and floral aromas, include methyl salicylate, benzyl alcohol, phenylethyl alcohol, and benzaldehyde [28,29]. The production of benzyl alcohol and phenylethyl alcohol is associated with plant stress responses, including *E. onukii* infestation [4]. In this study, the contents of benzyl alcohol and phenylethyl alcohol increased as withering progressed. Previous research has also shown that the levels of methyl salicylate, benzyl alcohol, and phenylethyl alcohol increase with extended withering time [30]. This suggested that these VCs are not only readily released from fresh leaves under *E. onukii* infestation, but also are significantly influenced by the withering process (leaf dehydration).

It was observed that the content of terpenoids (e.g., linalool, geraniol) increased significantly during withering, particularly when the moisture content was reduced to 55%. This phenomenon may be attributed to the synergistically enhanced activity of glycosidases (e.g., β-glucosidase) during leaf dehydration, which facilitates the hydrolysis of glycosidically bound terpenoid precursors into their volatile free forms [14,23]. Furthermore, infestation by *E. onukii* is known to upregulate the expression of terpene synthase genes (e.g., *CsTPS*) in tea plants [10], leading to a higher accumulation of terpenoid precursors prior to withering.

In summary, withering to 55% moisture content (17 h) is a critical stage for the formation of the aroma profile of Cicada black tea. Under this condition, the synergistic accumulation of terpenoids (e.g., geraniol, linalool) and esters (e.g., methyl salicylate, ethyl-2-(5-methyl-5-vinyltetrahydrofuran-2-yl)propan-2-yl carbonate), along with the optimized ratio of hydrocarbons to alcohols, collectively shape the intense floral, honey-like, and sweet aroma characteristics of Cicada black tea. Additionally, six VCs—geraniol, linalool, (z)-linalool oxide (furanoid), indole, dehydrolinalool, and phenylethyl alcohol—demonstrate regular and significant changes during withering. These compounds can serve as marker VCs to monitor the withering process during Cicada black tea production.

### 3.4. Comparative Analysis of Volatile Compounds in Cicada Black Tea Under Different Fermentation Conditions

To determine the regulatory effects of fermentation time on the aroma formation of Cicada black tea, this study employed a PLS-DA model to analyze the differences in VCs among tea samples subjected to three fermentation durations (3, 5, and 7 h).

**Figure 3:**
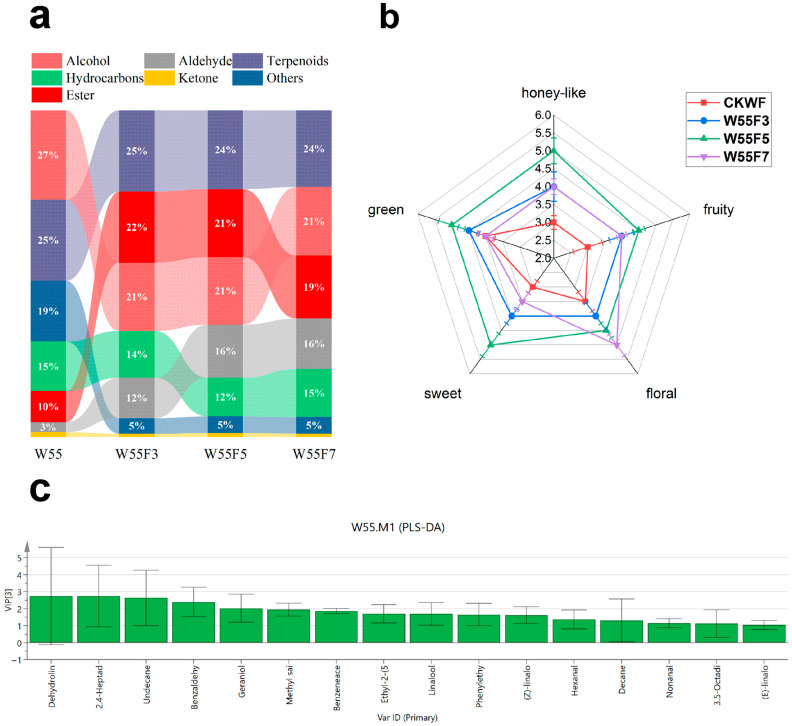
Screening and analysis of representative volatile components during the fermentation process of Cicada black tea. (**a**) Dynamic changes in volatile compounds in gas chromatography–mass spectrometry chromatograms across seven processing stages from W55 to W55F7. (**b**) Aroma radar chart combining odor activity values (OAVs) and sensory evaluation. (**c**) Sixteen key volatile compounds with variable importance in projection (VIP) > 1. (W followed by numbers indicates withering degree, i.e., leaf moisture percentage; F followed by numbers denotes withering duration in hours). PLS-DA, Partial Least Squares Discriminant Analysis.

The PLS-DA results revealed significant separation in the metabolic profiles of different fermentation treatment groups (R^2^Y = 0.955, Q^2^ = 0.897), indicating that fermentation duration had a decisive influence on the dynamic evolution of aroma components (Figure S7). As shown in Figure S8, all 200 permutated samples were distributed below the original model, with parameters (R^2^ = 0.199, Q^2^ = −0.446) demonstrating the strong robustness of the model. The scores exhibited distinct spatial separation during the fermentation process. Specifically, withered leaves were located in the second and third quadrants, samples fermented for 3 h were positioned in the fourth quadrant, and samples fermented for 5 and 7 h were clustered closely in the first quadrant. The closer the spatial distance, the smaller the changes in volatile metabolites. Therefore, significant changes in VCs occurred between the withering and fermentation stages. With increasing fermentation time, the relative proportions of terpenoids and aldehydes increased significantly, while the relative proportions of other compounds and alcohols gradually decreased (Figure 3a). This trend was directly related to the enhanced activity of oxidative enzymes (e.g., polyphenol oxidase, lipoxygenase) during fermentation [8,31,32].

Among the 75 VCs identified, 16 compounds had variable importance in projection (VIP) values greater than 1 (Figure 3c). Linalool and its oxides made particularly significant contributions to the aroma profile of Cicada black tea. Dehydrolinalool exhibited significant dynamic changes during processing, with the highest VIP values during both the withering (3.33) and fermentation (2.74) stages. Linalool and (Z)-linalool oxide (furanoid) also had VIP values greater than 1 across different withering and fermentation treatments. Previous studies have confirmed that the contents of linalool and linalool oxides are profoundly influenced by *E. onukii* infestation [33] and are identified as key components of the aroma profile of Cicada black tea. Their low odor thresholds (e.g., 0.006 mg/kg for linalool in water) contributed significantly to the floral and fruity characteristics of Cicada black tea.

Additionally, in regular black tea, indole was no longer detectable (via GC–MS) after fermentation. However, in Cicada black tea, indole persists to some extent during fermentation before dropping below the detection line. This phenomenon might be attributed to the overexpression of indole synthase genes *CsTSA* and *CsTSB2* induced by *E. onukii* infestation [18].

Table 1 lists all volatile compounds with calculable odor thresholds. Compounds with an ROAV > 1 are generally considered to contribute significantly to aroma, while those with ROAVs between 0.1 and 1 serve as aroma modifiers. Among them, carotenoid-derived volatiles (CDVs) exhibited the most prominent contributions. β-Damascenone reached an ROAV value of 34,457.81 after 5 h of fermentation (Table 1), with its honey-like and sweet characteristics forming the core aroma profile of Cicada black tea. The ROAV value of trans-β-ionone increased from 5717.61 (W55) to 24,710.41 (7 h) with prolonged fermentation, and its violet and woody notes further enriched the complexity of the aroma (Table 1). Studies suggest that CDV formation depends on the oxidative degradation of carotenoids during fermentation, and pest stress might accelerate this process by activating the lipoxygenase (LOX) pathway [9,34].

Both β-Damascenone (honey, sweet, woody, rose-like) and trans-β-ionone (violet, woody, floral, sweet, fruity) exhibited ROAVs exceeding 10,000, confirming their roles as the dominant aroma compounds in finished Cicada black tea. These compounds impart the distinctive honey-like and sweet notes that define the tea’s unique aroma profile. CDVs originate from various carotenoids, including β-carotene, α-carotene, phytoene, lutein, and lycopene, and are generated through enzymatic oxidative degradation, photooxidation, autoxidation, or thermal degradation during fermentation, pan-firing, and drying [34,35]. CDVs such as β-ionone, geranyl acetone, α-ionone, and β-Damascenone typically have a significant impact on tea aroma quality [9,10,15,36]. Moreover, CDVs often exhibit sweet, floral, or fruity characteristics [34]. Therefore, the marked increase in CDV levels likely enhanced the formation of sweet and floral notes in black tea. Figure 3b presents the aroma scoring plot combining ROAV data and the sensory evaluation results. The analysis indicated that a fermentation time of 5 h achieved the highest scores for sweet, honey-like, and fruity aromas. However, over-fermentation (7 h) enhanced floral and grassy notes, but led to a weakening of the honey-like aroma characteristics. At 3 h of fermentation, the aroma profile was relatively balanced, but not sufficiently intense, indicating under-fermentation of the Cicada black tea samples. The control group received the highest scores for grassy notes, followed by floral and sweet aromas, suggesting an excessive presence of grassy notes in its aroma composition.

Additionally, (E,E)-2,4-heptadienal, (Z)-linalool oxide (furanoid), theaspirane, and geraniol exhibited ROAVs exceeding 1000, contributing to the rich floral, fruity, and sweet aromas that significantly influenced the overall aroma of Cicada black tea. Furthermore, 11 VCs had ROAVs > 100, 17 VCs had ROAVs > 1, and 9 VCs had ROAVs > 0.1, collectively shaping the tea’s complex and layered aroma profile.

**Figure 4:**
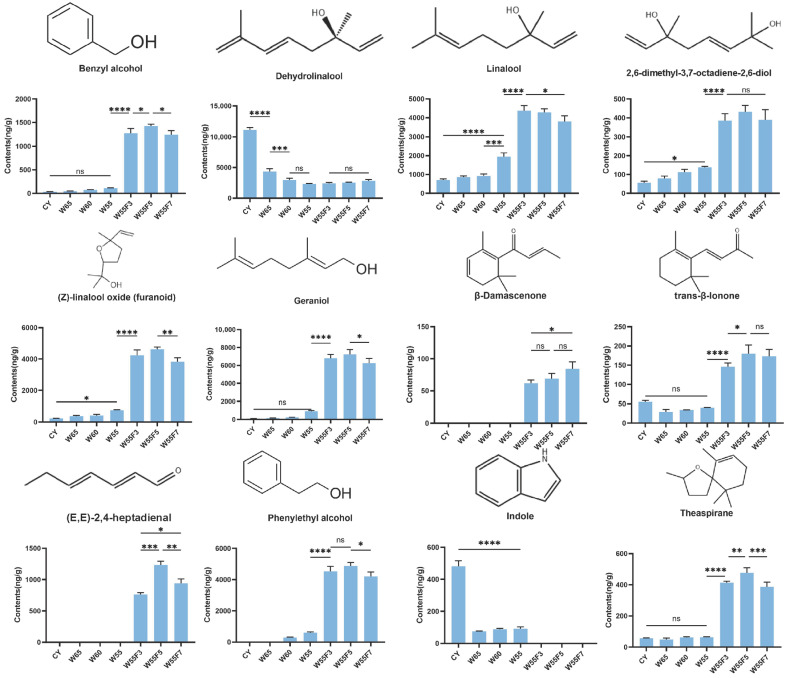
Dynamic changes in key volatile compounds during the processing of Cicada black tea. CY: fresh leaves; W: withering degree (numbers indicate moisture content percentage); F: fermentation time (numbers indicate hours). Data are presented as the mean ± SD (*n* = 3). Statistical significance was determined using one-way analysis of variance (ANOVA) (* *p* < 0.05, ** *p* < 0.01, *** *p* < 0.001, **** *p* < 0.0001; ns: not significant).

Therefore, a fermentation duration of 5 h was identified as the optimal parameter to achieve the highest aroma quality in Cicada black tea. It should be noted that the optimal parameters identified in this study are specific to the Jinxuan cultivar under the experimental conditions; their applicability to other tea cultivars requires further investigation. By integrating the changes in VCs during processing and focusing on the honey-like and sweet aroma characteristics, we screened compounds with VIP > 1 and ROAV > 1 or those contributing significantly to the aroma of Cicada black tea (ROAV > 1000). We propose that β-Damascenone, trans-β-ionone, (E,E)-2,4-heptadienal, theaspirane, geraniol, indole, (Z)-linalool oxide (furanoid), linalool, benzyl alcohol, phenylethyl alcohol, dehydrolinalool, and 2,6-dimethyl-3,7-octadiene-2,6-diol are the key volatile compounds in Cicada black tea processing (Figure 4). These compounds demonstrated regular and significant changes during the processing of Cicada black tea and are suitable to indirectly reflect biochemical transformations during its production.

## 4. Conclusions

This study determined the dynamic changes in VCs during the withering and fermentation processes of Cicada black tea, revealing the key factors contributing to its unique honey-like, sweet, and floral aroma. Ninety VCs were identified, among which 12 key compounds—β-Damascenone, trans-β-ionone, (E,E)-2,4-Heptadienal, Theaspirane, Geraniol, Indole, (Z)-linalool oxide (furanoid), Linalool, Benzyl alcohol, Phenylethyl alcohol, Benzaldehyde, and 2,6-Dimethyl-3,7-octadiene-2,6-diol—were identified as the primary contributors to its honey-like, floral, and fruity characteristics. Withering significantly increased the content of terpenoids and alcohols, thereby enhancing floral and fruity aromas, while fermentation elevated the levels of CDVs, such as β-Damascenone, which dominated the sweet and honey-like fragrance. The optimal processing conditions (withering to a moisture content of 55% and fermenting for 5 h) maximized the concentration of these unique honey-like and sweet aromas. Additionally, building upon existing literature, our findings align with the consensus that herbivore stress significantly influences the biosynthesis, degradation, and transformation of terpenoids in tea leaves, thereby shaping their aroma profiles. The experimental results presented in this study corroborate this conclusion, collectively elucidating the biochemical basis of the distinct flavor characteristics of cicada black tea. Thus, providing practical strategies to optimize the processing parameters (e.g., moisture control and fermentation time) to enhance the aroma quality and economic value of tea production from *E. onukii*-infested leaves.

## Figures and Tables

**Table 1:** Key volatile compounds responsible for the aroma profile of Cicada black tea and their relative odor activity values (ROAVs).

Chemical Name	CAS	Odor Perception ^A^	OT (μg/L in Water) ^B^	ROAVs			
				W55F3	W55F5	W55F7	CK-WF
β-Damascenone	23726-93-4	Honey, sweet, woody, rose	0.002	30,822.227	34,457.814	42,096.166	29,391.820
trans-β-ionone	79-77-6	Violet, woody, floral, sweet, fruity	0.007	20,879.506	25,719.719	24,710.415	21,688.007
(E,E)-2,4-heptadienal	4313-03-5	Fatty, nutty, violet, watermelon, grassy	0.36	2113.433	3420.378	2613.235	883.707
Theaspirane	36431-72-8	Black tea aroma, sweet with fruity/woody notes	0.2	2065.458	2380.895	1933.937	1661.464
Geraniol	106-24-1	Rose	6.6	1032.068	1097.773	949.199	559.839
Nerol oxide	1786-08-9	Floral, fatty	0.1	636.435	758.769	737.314	370.416
(Z)-linalool oxide (furanoid)	5989-33-3	Sweet, herbal	6	706.690	770.043	639.028	336.771
Linalool	78-70-6	Floral, sweet, rose	6	730.367	713.438	634.324	654.940
Benzeneacetaldehyde	122-78-1	Strong hyacinth with green leafy freshness	4	520.422	710.579	598.542	572.910
β-cyclocitral	432-25-7	Sweet, herbal, hay-like, fruity	0.3	221.486	275.750	241.522	234.115
Nerol	106-25-2	Rose and orange blossom	1.1	178.276	197.263	168.435	110.020
(E)-oct-2-enal	2548-87-0	Cucumber, vanilla, banana leaf	0.3	65.403	159.113	142.229	nd
Nonanal	124-19-6	Fresh, orange peel	1.1	89.569	167.858	140.647	573.318
2-pentylfuran	3777-69-3	Beany, fruity, earthy, green, vegetable-like	6	84.767	112.643	93.924	60.382
Phenylethyl alcohol	60-12-8	Rose, honey	45	100.655	108.493	93.394	46.175
Methyl salicylate	119-36-8	Wintergreen, minty	40	108.395	107.227	90.124	39.749
Safranal	116-26-7	Herbal, woody, saffron	0.3	74.040	nd	83.028	105.119
(Z)-3,7-Dimethyl-3,6-octadien-1-ol	5944-20-7	Sweet, floral	1.1	42.493	63.299	50.552	17.416
1-Hexanol	111-27-3	Fresh, green leaf, fruity	5.6	53.114	55.762	48.015	36.646
Hexanal	66-25-1	Grassy with apple	4.5	24.628	49.971	47.655	21.744
Dehydrolinalool	29957-43-5	With herbal floral aroma and spicy lavender-like fragrance	100	24.192	25.459	28.300	19.392
o-Cymene	527-84-4	Fatty aroma	4	19.174	21.714	26.912	19.645
Benzyl alcohol	100-51-6	Sweet, floral, clover, honey	100	12.696	14.235	12.482	5.733
(Z)-3-Hexenyl butanoate	16491-36-4	Fresh, sweet, fruity	31	7.785	7.611	5.937	3.369
D-Limonene	5989-27-5	Lemon, sweet orange peel, licorice	10	nd	5.471	5.587	5.051
(Z)-6,10-Dimethyl-5,9-undecadien-2-one	3879-26-3	With lemongrass aroma and slight medicinal notes	10	5.652	7.350	5.544	2.816
(E)-hex-3-en-1-ol	928-97-2	Grassy	110	6.438	5.950	4.838	3.155
(E)-linalool oxide (pyranoid)	39028-58-5	Woody	320	4.738	5.321	4.821	2.893
Benzaldehyde	100-52-7	Almond odor, caramel sweetness	750	3.844	5.251	4.670	2.781
(E)-oct-2-enal	928-95-0	Green leaf, mung bean, herbal, narcissus	100	5.158	4.797	3.843	3.918
Sulcatone	110-93-0	Fresh green with citrus notes	68	2.187	2.738	2.113	1.258
β-homocyclocitral	472-66-2	Fishy/salty, green	19	1.231	1.534	1.514	1.475
(Z)-hex-3-en-1-yl acetate	31501-11-8	Rose, lavender, apple	200	1.629	1.703	1.487	0.690
(E,E)-3,5-octadien-2-one	30086-02-3	Grassy	100	1.493	2.097	1.458	0.776
Geranial	141-27-5	Floral, orange, sweet	460	0.273	0.378	0.324	0.122
1-Octanol	111-87-5	Sweet waxy	190	0.350	0.384	0.297	nd
α-Terpineol	98-55-5	With pinewood and clove-like aroma	300	0.272	0.293	0.266	0.224
Benzyl-nitrile	140-29-4	Woody, balsamic, anise	1200	0.196	0.205	0.184	0.082
E-Nerolidol	40716-66-3	Orange blossom, floral	250	0.115	0.113	0.108	0.059
(Z)-hex-3-en-1-yl acetate	3681-71-8	Grassy	31	1.870	nd	nd	nd

Note: Odor thresholds (OTs) and odor characteristics were referenced from: ^A^ https://www.thegoodscentscompany.com (accessed on 10 March 2024) ^B^ https://www.femaflavor.org/flavor-library, https://www.thegoodscentscompany.com (accessed on 15 April 2024), nd: The compounds were not detected in the sample.

## Data Availability

Data is contained within the article.

## Supplementary Materials

The following supporting information can be downloaded at: https://www.mdpi.com/article/10.3390/foods15020401/s1, Figure S1: Score plots of volatile components derived from GC-MS analysis of fresh leaves of CY and CK species by OPLS-DA; Figure S2: Validation of the OPLS-DA model of the dynamic non-volatile compounds in CY and CK. Figure S3: The score plots of the volatile components in Cicada black tea from GC–MS during the processing stages CKWF and W55F5 by OPLS-DA; Figure S4: Validation of the OPLS-DA model of the dynamic non-volatile compounds in CKWF and W55F5; Figure S5:The score plots of the volatile components in Cicada black tea from GC–MS during the processing stages from CY to W55 by PLSDA; Figure S6: Validation of the PLSDA model of the dynamic non-volatile compounds from CY to W55; Figure S7: The score plots of the volatile components in Cicada tea from GC–MS during the processing stages from W55 to W55F7 by PLSDA; Figure S8: Validation of the PLSDA model of the dynamic non-volatile compounds from W55 to W55F7. Table S1: Relative content of tea leaves samples classified into seven categories; Table S2: Key volatile compounds responsible for the aroma profile of Cicada black tea withered leaves and their relative odor activity values (ROAVs).
