# Supplementary material for: Influence of Key Processes on the Aroma Formation of Cicada Black Tea
Source: Foods. 2026 Jan 22;15(2):401. doi: 10.3390/foods15020401 (PMC12841396; doi:10.3390/foods15020401)
Supplement: Supplementary file 1 [file foods-15-00401-s001.zip › foods-4072922-supplementary.pdf]

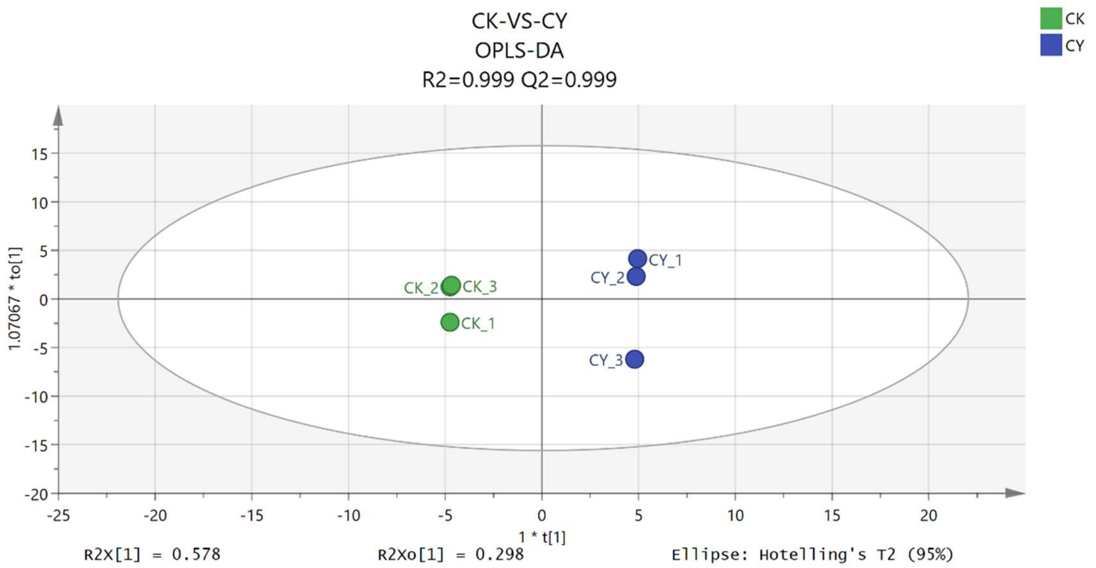

Fig.S1 Score plots of volatile components derived from GC-MS analysis of fresh leaves of CY and CK species by OPLS-DA

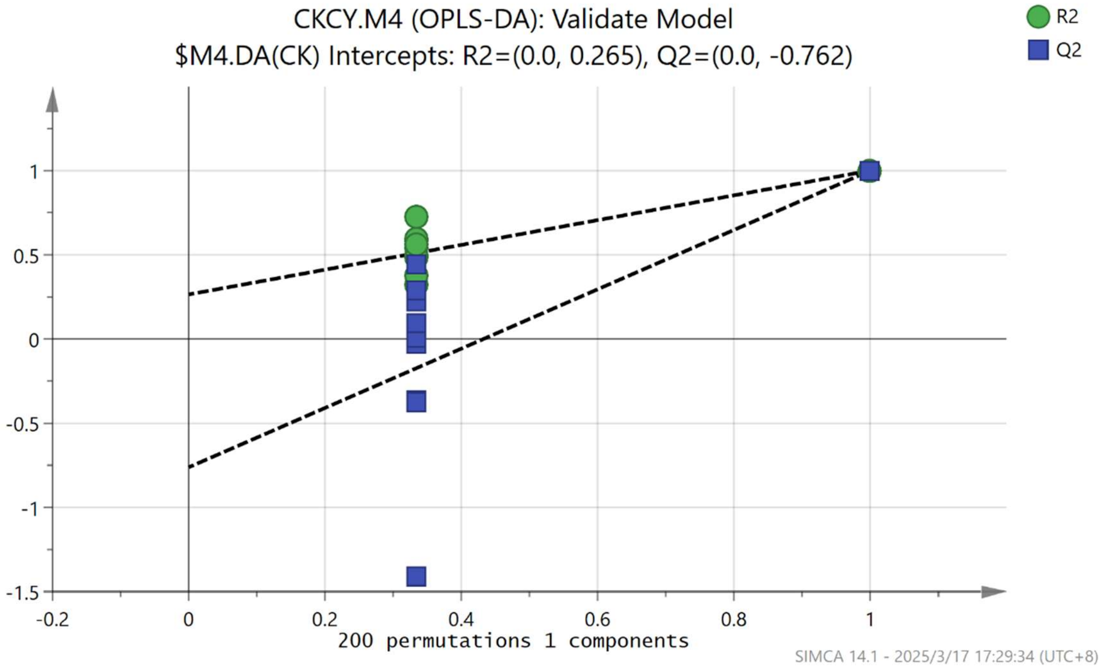

Fig.S2 Validation of the OPLS-DA model of the dynamic non-volatile compounds in CY and CK

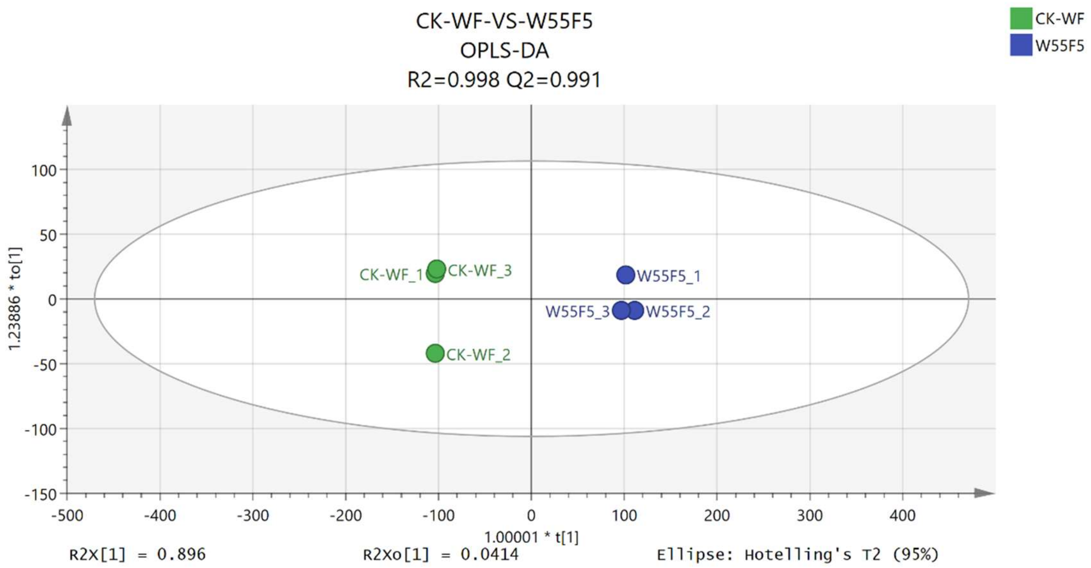

Fig.S3 The score plots of the volatile components in Cicada black tea from GC–MS during the processing stages CKWF and W55F5 by OPLS-DA

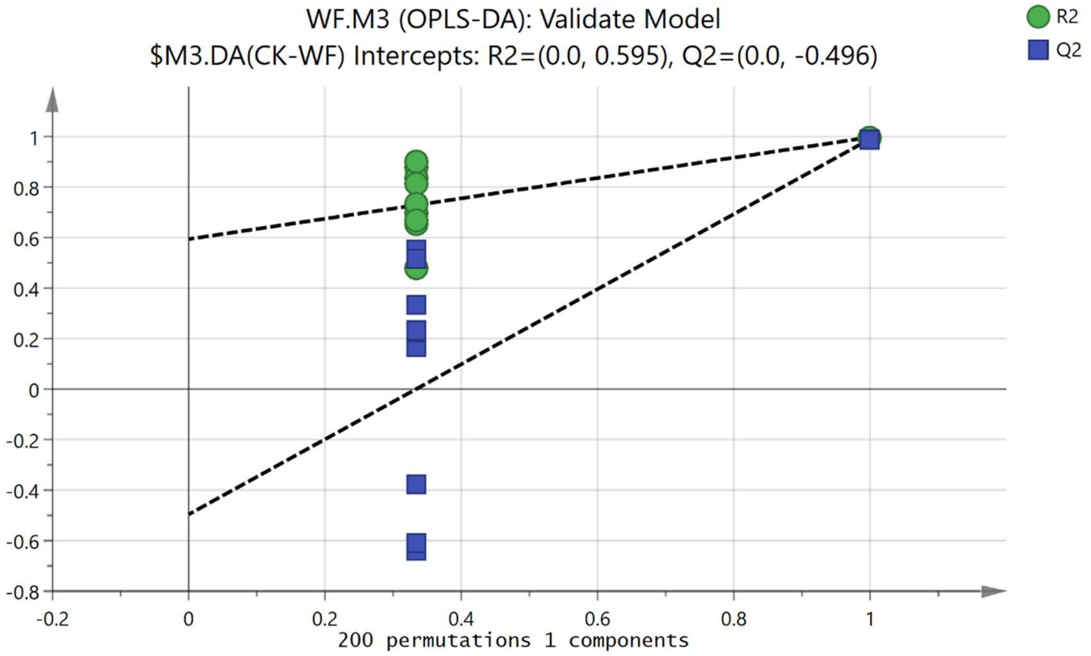

Fig.S4 Validation of the OPLS-DA model of the dynamic non-volatile compounds in CKWF and W55F5

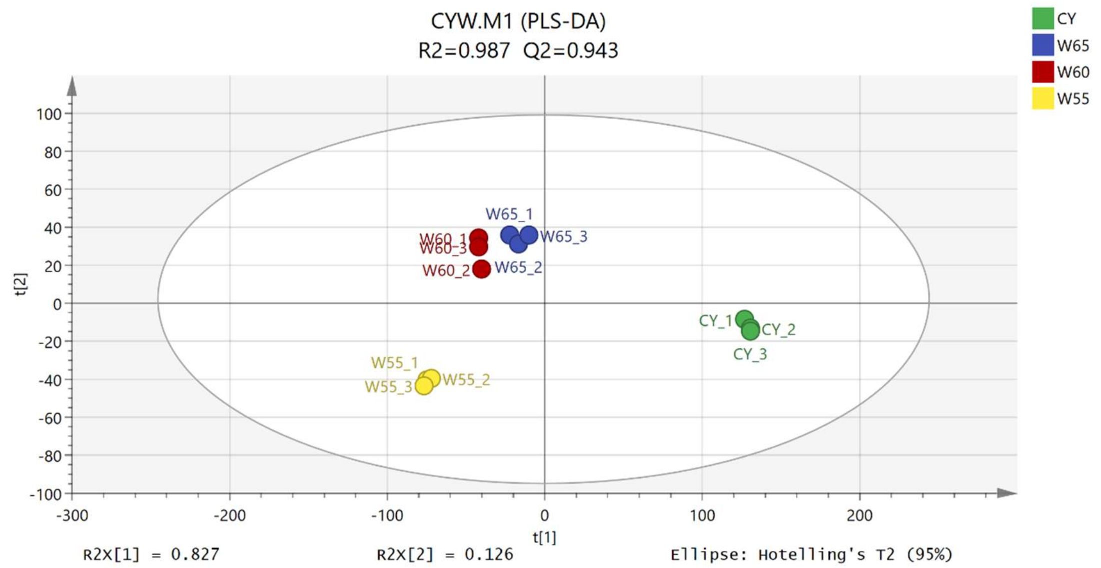

Fig.S5 The score plots of the volatile components in Cicada black tea from GC–MS during the processing stages from CY to W55 by PLSDA

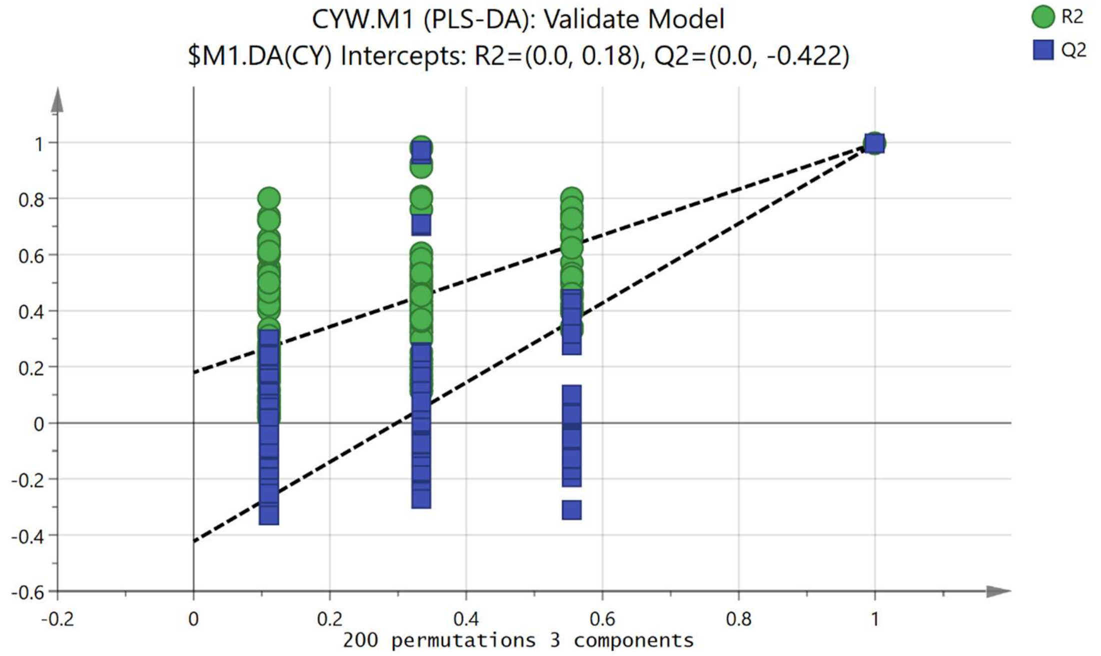

Fig.S6 Validation of the PLSDA model of the dynamic non-volatile compounds from CY to W55

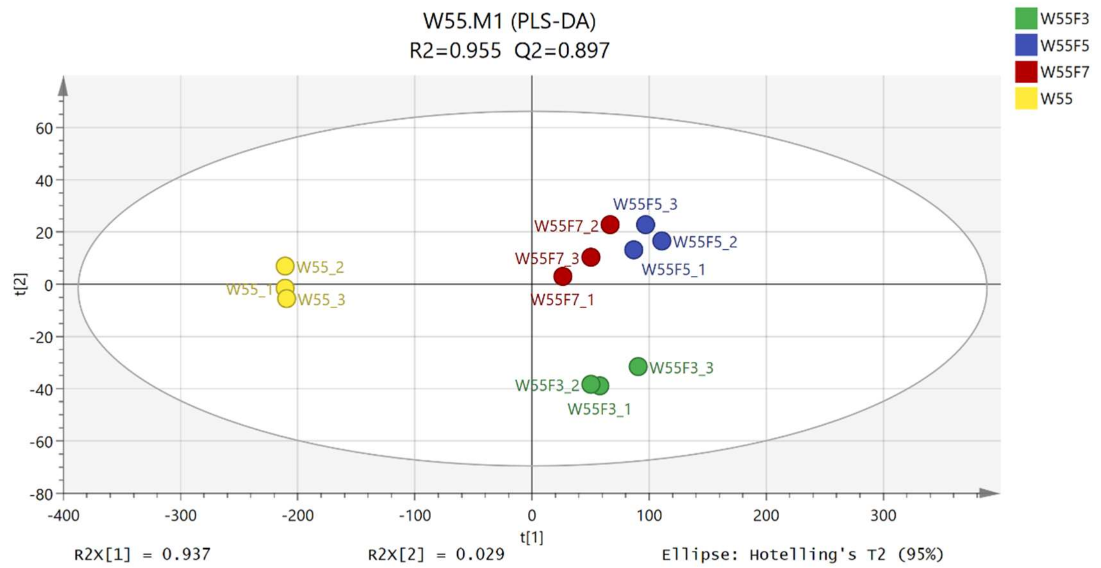

Fig.S7 The score plots of the volatile components in Cicada tea from GC–MS during the processing stages from W55 to W55F7 by PLSDA

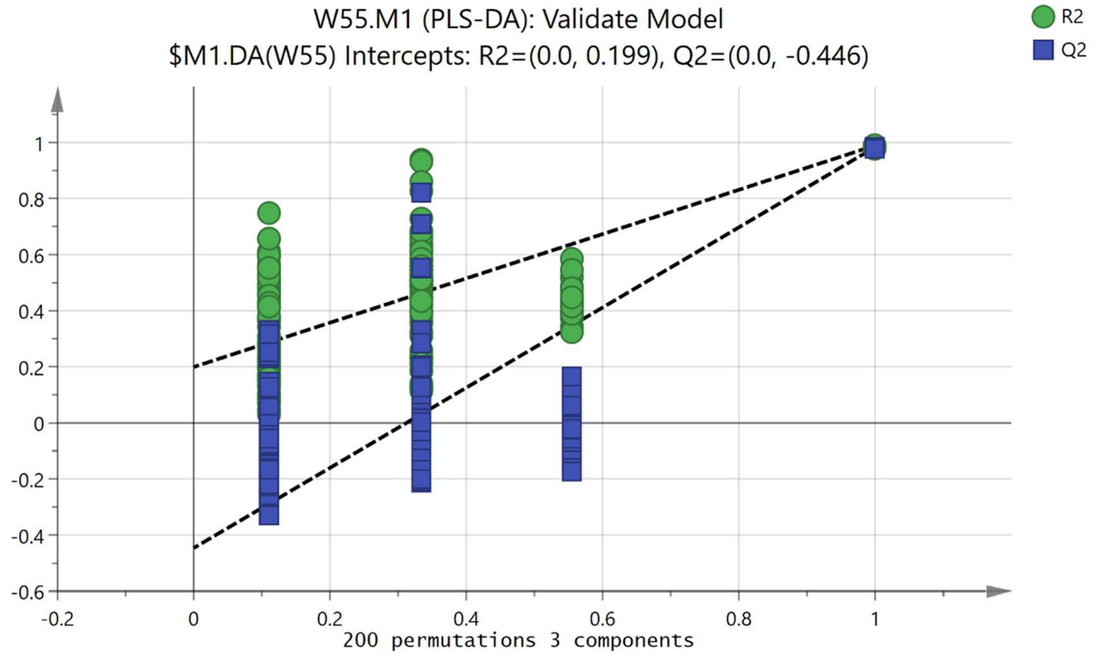

Fig.S8 Validation of the PLSDA model of the dynamic non-volatile compounds from W55 to W55F7

Table S1.

Relative content of tea leaves samples classified into seven categories.

NO	RI (ref)	RI (cal)	Chemical name	relative content(ng/g)								
				CK-WF	CK	CY	W65	W60	W55	W55F3	W55F5	W55F7
				Terpenoids								
1	988	991	β-Myrcene	-	598.9±8.99	682.12±69.21	-	-	-	-	-	-
2	1018	1017	α-terpinene	-	164.53±2.15	173.64±16.75	-	-	-	-	-	-
3	1030	1031	D-Limonene	50.51±1.67	417.78±5.13	483.04±53.4	89.4±18.79	62.39±6.68	44.47±9.36	-	54.71±1.59	55.87±7.18
4	1034	1038	(Z)-β-ocimene	-	162.05±7.16	200.98±21.56	-	-	-	-	-	-
5	1086	1088	Terpinolene	-	-	193.93±18.08	-	-	-	-	-	-
6	1098	1099	Linalool	3929.64±360.18	699.93±28.27	703.08±66.49	863.86±57.54	919.64±105.4	1952.73±195.81	4382.2±267.42	4280.63±196.14	3805.94±293.88
7	1139	1131	(E,Z)-alloocimene	-	-	78.42±10.45	-	-	-	-	-	-
8	1195	1189	α-Terpineol	67.12±7.76	63.57±2.3	86.68±9.13	37.91±4.89	28.6±3.5	33.98±4.69	81.64±4.53	87.88±5.71	79.93±6.09
9	1220	1220	β-cyclocitral	70.23±6.15	35.18±1.1	38.17±2.33	31.67±7.23	33.14±2.94	33.1±1.95	66.45±6.22	82.72±4.73	72.46±5.62
10	1221	1228	Nerol	121.02±11.65	-	-	-	-	-	196.1±8.01	216.99±12.25	185.28±17.85
11	1247	1255	Geraniol	3694.94±349.16	55.75±4.97	103.29±5.47	152.89±8.52	231.77±18.51	932.9±69.31	6811.65±399.42	7245.3±526.32	6264.71±517.12

12	1302	1302	Theaspirane	332.29±44.75	43.03±1.2	56.95±2.53	49.7±8.72	62.28±4.44	63.4±3.4	413.09±9.86	476.18±33.12	386.79±30.64
13	1377	1386	β-Damascenone	58.78±8.86	-	-	-	-	-	61.64±5.18	68.92±8.3	84.19±11.05
14	1476	1486	trans-β-lonone	151.82±7.18	50.82±3.2	55.23±3.21	29.3±6.28	34.08±0.13	40.02±0.89	146.16±9.58	180.04±22.32	172.97±17.87
15	1519	1518	β-cadinene	60.31±9.03	46.99±11.56	63.42±10.46	33.42±1.79	45.71±7.27	66.01±5.65	70.22±2.27	67.18±8.55	73.74±4.05
16	1523	1529	trans-calamenene	40.48±5.68	42.26±9.42	64.02±7.37	26.79±2.04	37.16±6.63	47.12±3.07	57.28±2.43	58.11±9.55	60.98±1.68
17	1544	1542	α-Calacorene	16.41±1.39	15.95±3.14	28.06±3.26	8.13±0.38	8.79±1.75	11.53±1.2	21.72±0.92	20.93±2.13	24.06±1.38
18	1556	1564	E-Nerolidol	14.77±1.11	-	-	-	-	-	28.86±3.68	28.35±6.29	27±2.77
Others												
19	725.5	731	Butanenitrile.3-melthyl-	-	-	275.67±36.1	-	-	-	-	-	-
20	910	917	Pyrazine.2.6-dimethyl-	-	217.1±12.39	138.61±20.23	-	-	-	-	-	-
21	958	964	5-Methyl furfural	-	-	88.09±3.5	-	-	-	-	-	-
22	989	993	Furan.2-penthyl-	362.29±50.7	-	-	189.04±21.1	231.32±41.42	238.15±2.49	508.6±14.79	675.86±81.56	563.54±83.71
23	1000	1003	Pyrazine.2.	-	182.12±6.64	108.34±0.55	-	-	-	-	-	-

			-ethyl-6-methyl-									
24	1047	1046	Tea pyrrole	78.36±3.23	1067.82±52.92	905.87±60.72	82.23±9.72	40.27±12.37	-	-	-	-
25	1059	1063	Ethanone.1-(1H-pyrrol-2-yl)-	-	-	340.25±17.63	-	-	-	-	-	-
26	1135	1144	Benzyl-nitrile	98.4±9.6	195.48±10.03	395.59±27.98	144.12±25.95	115.14±12.61	164.7±13.09	234.9±20.32	245.52±17.54	221.36±17.29
27	1150	1154	Nerol oxide	37.04±4.73	-	83.69±2.67	-	-	16.71±0.73	63.64±5.42	75.88±3.51	73.73±8.39
28	1173	1173	(E)-linalool oxide (pyranoid)	925.91±97.81	54.85±6.77	86.4±2.25	120.13±9.41	178.52±25.44	264.81±8.8	1516.24±74.25	1702.59±73.31	1542.84±80.35
29	1290	1295	Indole	-	381.47±6.31	482±33.88	75.5±1.86	88.25±5.11	91.67±11.6	-	-	-
						Ketone						
30	982	986	Sulcatone	85.55±8.29	106.8±3.66	119.58±4.82	147.59±20.26	139.79±32.23	121.64±9.87	148.73±12.14	186.18±10.46	143.69±22.18
31	1067	1073	3.5-Octadien-2-one.(E.E)-	77.62±6.87	-	-	-	-	-	149.26±13.01	209.68±8.54	145.82±9.84
32	1139	1144	(R.S)5-ethyl-6-methyl-3e-hepten-2-one	33.28±3.17	-	-	21.39±3.9	36.1±3.63	44.5±2.46	87.29±5.38	114.67±9.74	94.8±13.84

33	1282	1283	3-Undecanone	40.68±5.92	-	-	-	-	-	34.37±3.8	21.82±3.68	40.63±2.51
34	1442	1435	5.9-Undecadien-2-one-6-10-dimethyl-.(Z)-	28.16±1.88	-	34.66±5.17	24.38±6.33	18.71±2.72	20.66±1.06	56.52±3.73	73.5±13.34	55.44±6.02
						Hydrocarbons						
35	997	993	2.6-Dimethyl-2-tans-6-octadinene	-	287.26±16.28	286.7±35.6	-	-	-	-	-	-
36	999	1000	Decane	244.1±60.53	54.02±0.72	-	62.05±19.43	97.53±3.72	80.81±10.83	193.92±26.93	117.5±38.37	198.43±42.36
37	1021	1013	Benzene.1.2.3-trimethyl-	-	52.65±0.22	55.47±2.55	-	-	-	-	-	-
38	1025	1022	o-Cymene	78.58±12.59	472.99±16.71	789.88±29.52	161.69±26.26	100.92±14.31	61.24±0.73	76.7±3.03	86.85±10.12	107.65±17.63
39	1045	1048	α-Ocimene	123.44±13.58	477.64±15.55	544.92±93.12	153.02±41.11	103.98±9.94	97.84±12.55	291.39±11.41	278.69±26.18	245.44±42.04
40	1063	1064	Decane.2-methyl-	62.55±11.28	-	-	27.48±2.4	35.31±2.18	29.45±2.48	-	-	-
41	1072	1074	(Z)-linalool oxide	2020.63±169.48	135.98±17.57	230.1±10.4	377.3±42.89	424.52±68.48	749.28±36.37	4240.14±347.56	4620.26±144.44	3834.17±254.21

			(furanoid)									
42	1090	1082	Benzene.1-methyl-3-(1-methylethenyl)-	108.77±21.34	477.39±27.15	783.49±79.01	147.44±8.05	92.59±7.74	73.67±6.03	108.59±4.78	116.71±11.19	134.03±14.37
43	1098	1100	Undecane	1542.6±338.94	265.58±14.13	246.92±41.96	455.53±95.18	412.53±49.21	366.33±39.83	1416.11±86.51	-	1455.43±37.85
44	1111	1116	(E)-4.8-Dimethylnona-1.3.7-triene	-	381.84±33.19	485.75±151.35	231.24±83.17	198.2±11.23	181.57±22.42	-	344.35±34.85	303.01±89.63
45	1120	1119	para-menthatriene	-	31.11±2.46	58.34±7.88	-	-	-	-	-	-
46	1126	1131	cis-Allo-ocimene	27.99±3.28	146.2±6.29	198.27±27.52	27.14±6.56	17.26±1.86	17.33±2.62	41.92±0.53	39.83±2.39	40.91±3.48
47	1129	1131	cosmene	40.81±9.29	222.18±18.6	610.55±74.31	131.71±25.71	73.41±1.95	41.01±1.88	40.05±0.86	34.19±1.24	51.74±2.11
48	1153	1156	Undecane. 5-methyl-	29.45±5.73	-	-	8.32±0.23	9.76±1.39	7.77±1.33	23.36±1.65	16.47±3.53	25.66±3.76
49	1186	1178	1-Methoxyadamantane	-	64.54±10.91	85.84±17.7	-	-	-	-	-	-
50	1188	1182	Naphthalene	-	58.34±0.79	56.89±3.26	-	-	-	-	-	-

51	1197	1200	Dodecane	392.86±87.07	-	110.73±29.22	169.58±22.2	109.87±7.86	86.29±11.85	336.45±20.65	311±11.41	343.31±54.2
52	1356	1354	Naphthalene.1.2-dihydro-1.1.6-trimethyl-	34.77±5.57	58.49±9.05	67.44±6.8	10.86±1.52	-	3.57±0.2	12.94±0.82	13.29±1.29	24.68±2.37
53	1366	1371	Tridecane.3-methyl-	109.17±12.16	38.38±11.38	66.47±16.95	66.58±10.63	38.12±5.63	37±4.27	92.58±9.56	76.55±11.86	99.12±8.23
54	1395	1400	Tetradecane	140.52±18.72	82.9±23.92	122.68±32.02	99.98±11.04	82.86±10.2	76.28±3.8	125.9±7.78	130.19±11.27	125±10.55
55	1447	1452	Cyclopentane.nonyl-	17.89±2.1	9.23±1.5	11.07±2.31	10.07±1.72	6.6±1.45	6.06±1.08	20.31±1.14	19.8±1.9	21.25±3.54
56	1455	1449	2.6.10-Trimethyltridecane	22.85±2.55	10.9±4.41	-	-	9.74±2.14	11.45±0.77	19.67±2.94	21.7±3.62	-
57	1494	1500	Pentadecane	-	18.98±6.08	15.12±3.77	-	-	8.63±0.03	18±3.56	22.92±4.74	-
58	1565	1570	Pentadecane.3-methyl-	28.17±1.2	37.93±8.06	50.42±9.05	22.43±3.57	16.81±5.66	19.98±0.44	24.34±0.12	23.01±2.44	24.06±4.95
59	1594	1600	Hexadecane	28.11±1.83	28.58±4.66	33.27±6.29	11.81±2.59	12.92±4.38	13.35±0.28	29.18±4.05	35.96±7.78	17.23±3.26
							Ester					
60	1002	1006	3-Hexen-1-ol,acetate.(Z)-	-	-	-	38.23±7.49	59.55±20.87	54.59±8.38	57.97±5.49	-	-

61	1087	1090	Ethyl-2-(5-methyl-5-vinyltetrahydrofuran-2-yl)propan-2-yl carbonate	2494.96±209.64	103.18±0.92	136.47±5.83	254.15±14.52	398.04±68.17	727.12±6.21	4694.43±351.02	5105.02±137.54	4249.96±263.76
62	1181	1187	Butanoic acid.3-hexenyl ester.(Z)-	104.44±16.53	-	-	39.98±3.63	77.26±9.23	57.05±6.07	241.33±15.83	235.95±17.66	184.05±17.22
63	1189	1195	Butanoic acid.2-hexenyl ester.(E)-	123.9±22.84	-	-	13.61±5.23	-	26.62±1.94	218.13±11.83	219.17±22.21	167.85±18.98
64	1192	1192	Methyl sailcylate	1589.95±184.37	153.46±5	340.27±18.94	107.82±11.62	-	216.66±13.23	4335.8±200.27	4289.06±290.04	3604.96±230.45
65	1226	1234	cis-3-Hexenyl-α-methylbutyrate	-	-	-	-	46.04±5.46	48.9±3.92	188.36±16.09	190.29±11.85	154.16±18.92
66	1232	1238	cis-3-Hexenyl isovalerate	87.36±17.18	-	-	-	-	-	193.38±20.28	207.81±12.81	168.02±21.87
67	1373	1380	Hexanoic	137.93±18.09	-	-	71.12±2.85	101.98±9.82	59.43±6.18	325.82±20.38	340.52±42.4	297.39±18.03

			acid.3-Hexenyl ester.(Z)-									
68	1379	1384	Hexanoic acid.hexyl ester	-	-	-	-	17.29±2.36	15.27±1.66	51.26±2.85	53.04±9.93	48.34±3
69	1381	1391	Hexanoic acid.2-Hexenyl ester.(E)-	102.95±12.9	-	-	-	27.93±2.04	23.31±1.45	190.78±11.52	213.12±32.26	181.31±10.28
70	1407	1410	Methyl-4-tert-butylbenzoate	9.17±0.88	-	-	-	-	-	16.54±0.92	18.29±2.9	16.38±1.33
71	1583	1588	2.2.4-Trimethyl-1.3-pentanediol diisobutyrate	183.98±9.03	-	-	-	-	-	263.4±14.38	256.44±63.1	203.71±39
						Aldehyde						
72	799	801	Hexanal	97.85±6.8	-	-	-	-	-	110.83±2.86	224.87±5.29	214.45±25.19
73	961	962	Benzaldehyde	2085.81±192.91	85.49±3.73	154.02±9.54	143.55±18.12	179.39±16.75	272.56±11.72	2882.69±205.47	3938.49±157.34	3502.57±370.11
74	1009	1012	2.4-	318.13±31.58	-	-	-	-	-	760.84±30.23	1231.34±60.34	940.76±69.48

			Heptadienal.(E.E)-									
75	1043	1045	Benzeneacetaldehyde	2291.64±142.38	-	-	-	73.41±10.02	101.88±7.58	2081.69±257.61	2842.32±149.59	2394.17±213.37
76	1056	1060	2-Octenal.(E)-	-	-	-	-	-	-	19.62±0.76	47.73±3.06	42.67±6.89
77	1102	1104	Nonanal	630.65±944.47	-	-	-	-	-	98.53±10.1	184.64±6.93	154.71±12.26
78	1199	1201	Safranal	31.54±1.9	22.85±3.74	33.55±3.23	20.52±3.43	13.82±0.63	11.31±0.74	22.21±0.29	-	24.91±0.56
79	1256	1254	beta-Homocyclocitral	28.03±2.26	-	-	-	-	-	23.39±1.55	29.15±1.47	28.77±1.84
80	1264	1270	Geranial	55.91±4.71	-	-	-	-	-	125.69±7.3	174±13.78	149.04±10.27
						Alcohol						
81	852	852	3-Hexen-1-ol,(E)-	347.06±26.27	-	-	-	52.16±14	94.49±14.58	708.14±66.6	654.54±34.51	532.23±55.46
82	862	862	2-Hexen-1-ol,(E)-	391.78±29.87	-	-	-	-	49.59±12.41	515.76±50.29	479.71±29.98	384.29±29.35
83	866	868	1-Hexanol	205.22±17.01	-	-	-	55.61±19.89	104.15±19.03	297.44±58.96	312.27±32.74	268.88±27.73
84	1032	1036	Benzyl alcohol	573.31±53.33	-	34.48±2.98	45.38±3.08	72.44±9.07	110.58±6.23	1269.57±101.19	1423.45±40.97	1248.2±79.3
85	1068	1070	1-Octanol	-	-	-	25.44±0.91	-	36.35±3.43	66.42±7.1	73.03±2.75	56.39±9.32
86	1101	1107	Dehydrolinalool	1939.24±174.86	5505.71±185.43	11084.25±391.86	4344.95±482.19	2959.94±286.09	2317.59±102.37	2419.16±128.76	2545.93±76.08	2829.98±216.98
87	1111	1116	Phenylethyl alcohol	2077.9±183.85	-	-	-	297.48±27.77	595.48±58.28	4529.47±318.17	4882.19±208.48	4202.72±282.99

88	1185	1193	3.7-Octadiene-2.6-diol.2.6-dimethyl-	148.73±11.16	-	56.12±7.96	78.86±12.58	112.66±14.81	138.69±4.46	385.08±36.96	432.08±33.92	388.81±54.65
89	1186	1183	cherry propanol	88.4±9.54	-	-	80.2±7.49	73.71±6.62	85.58±6.04	141.89±9.87	152.35±7.76	130.18±8.26
90	1235	1240	3.6-Octadien-1-ol.3.7-dimethyl-.(Z)-	19.16±0.25	-	-	-	-	-	46.74±2.49	69.63±4.63	55.61±10.26

Note: Chemical names and concentrations of compounds in aroma extract of Cicada black tea and control regular tea samples infusion (No: Detected volatile compound marker; RI(ref):
retention index; RI(cal): Response factor relative to internal standard.

Table S2.

Key volatile compounds responsible for the aroma profile of Cicada black tea withered leaves and their relative odor activity values (ROAVs).

NO	Name	CAS	Odor characteristics[A]	OT (mg/L in water)[B]	ROAVs		
					W65	W60	W55
1	Theaspirane	36431-72-8	Black tea aroma, sweet, with fruity and woody notes	0.2	nd	nd	83.56871694
2	Geraniol	106-24-1	Rose	6.6	28.64245525	35.04862364	36.08359231
3	Nerol	106-25-2	Rose and orange blossom	1.1	22.16157752	17.0055688	18.7812363
4	Linalool	78-70-6	Floral, sweet, rose	6	26.9484892	16.81964512	10.20630032
5	D-Limonene	5989-27-5	Lemon, sweet orange peel, licorice	10	3.823024787	5.954570676	5.458748421
6	(Z)-β-ocimene	3338-55-4	Apple	34	1.175794796	2.272230186	1.67786089
7	α-Terpineol	98-55-5	Pine and clove-like aroma	300	0.491958762	0.465955828	0.405482845
8	E-Nerolidol	40716-66-3	Orange blossom, floral	250	0.133675434	0.182833332	0.264052018
9	β-cadinene	523-47-7	Woody, hay	1500	0.033131478	0.041517566	0.04226676
10	α-terpinene	99-86-5	Citrus and lemon	300	0.068413144	0.046080798	0.037693102
11	Indole	120-72-9	Jasmine	0.04	3602.964482	2878.509044	4117.453057
12	Tea pyrrole	2167-14-8	Floral, rose, balsamic	3.7	nd	12.44358592	13.21738207
13	5-Methyl furfural	620-02-0	Spicy-sweet, warm, slightly caramel	6000	nd	0.049579613	0.09924685
14	Pyrazine.2.-ethyl-6-methyl-	13925-03-6	Roasted, nutty, grassy	1500	nd	0.048941546	0.067919593
15	Benzyl-nitrile	140-29-4	Woody, balsamic, anise	1200	0.109761744	0.061173325	0.034170862
16	(E)-linalool oxide	39028-58-	Woody	320	0.033941578	nd	0.011155934

	(pyranoid)	5					
17	Sulcatone	110-93-0	Fresh green with citrus notes	68	1.179343226	1.084009964	1.25857701
18	3.5-Octadien-2-one.(E.E)-	30086-02-3	Grassy	100	nd	0.279345616	0.233097474
19	para-menthatriene	18368-95-1	Turpentine, camphor, herbal woody	15	15.41598539	13.21361133	12.1046955
20	Naphthalene	91-20-3	pungent dry tarry	6	nd	nd	1.438414817
21	Naphthalene.1.2-dihydro-1.1.6-trimethyl-	30364-38-6	Licorice	900	0.133480587	0.19835033	0.294234702
22	Butanoic acid.3-hexenyl ester.(Z)-	16491-36-4	Refreshing, sweet, fruity	31	2.147716381	1.229675397	1.193483868
23	Methyl sailcylate	119-36-8	Wintergreen, mint	40	0.295291437	0.32293055	0.333808647
24	Hexanoic acid.hexyl ester	6378-65-0	Apple and pineapple-like aroma	6400	0.013968805	0.009748787	0.006948739
25	Geranial	141-27-5	Floral, orange, sweet	460	0.178769343	0.087546776	nd
26	Nonanal	124-19-6	Fresh, orange, zest	1.1	343.0029693	385.9308947	681.160126
27	Safranal	116-26-7	Herbal, woody, saffron	0.3	71.31380959	120.3454905	148.3259219
28	Hexanal	66-25-1	Grassy with apple aroma	4.5	nd	nd	11.01958624
29	Benzeneacetaldehyde	122-78-1	Strong hyacinth aroma and green leafy freshness	4	3.40198773	nd	6.654859964
30	Benzaldehyde	100-52-7	Almond scent, caramel sweetness	750	0.105149159	0.150212006	0.184913718
31	Benzyl alcohol	100-51-6	Sweet, floral, clover, honey	100	2.541539513	3.980433547	7.271239721
32	1-Hexanol	111-27-3	Refreshing, green leaves, fruity	5.6	4.54220817	nd	6.490259254
33	Phenylethyl alcohol	60-12-8	Rose, honey	45	3.276361577	2.057631673	1.637122273
34	2-Hexen-1-ol,(E)-	928-95-0	Green leaves, mung bean, herbal, narcissus	100	0.453805803	0.724435432	1.105834893
35	3-Hexen-1-ol,(E)-	928-97-2	Grassy	110	nd	0.505584225	0.946837011
36	Dehydrolinalool	29957-43-	Floral and herbal aroma with spicy and	100	0.274844869	0.353113214	0.294506657

	5	lavender-like notes

Note: Odor thresholds (OTs) and odor characteristics were referenced from:[A] https://www.thegoodscentscompany.com.[B] https://www.femaflavor.org/flavor-library, https://www.thegoodscentscompany.com

Nd: The compounds were not detected in the sample.
